# Averting an Impending Storm: Can We Reengineer Health Systems to Meet the Needs of Aging Populations?

**DOI:** 10.1371/journal.pmed.1001267

**Published:** 2012-07-17

**Authors:** Arlene S. Bierman

**Affiliations:** 1 Keenan Research Centre in the Li Ka Shing Knowledge Institute, St. Michael's Hospital, Toronto, Ontario, Canada; 2 Lawrence S. Bloomberg Faculty of Nursing; Institute of Health Policy, Evaluation and Management; Department of Medicine, University of Toronto, Toronto, Ontario, Canada

## Abstract

Arlene Bierman discusses new research findings from a randomized trial evaluating community-based nursing interventions in older adults, and and comments on how we need to to re-engineer health systems to provide greater quality of care.

**Linked Research Article**

This Perspective discusses the following new study published in *PLoS Medicine*:

Coburn KD, Marcantonio S, Lazansky R, Keller M, Davis N (2012) Effect of a Community-Based Nursing Intervention on Mortality in Chronically Ill Older Adults: A Randomized, Controlled Trial. PLoS Med 9(7): e1001265. doi:10.1371/journal.pmed.1001265

Kenneth Coburn and colleagues report findings from a randomized trial evaluating the effects of a complex nursing intervention on mortality risk amongst older individuals diagnosed with chronic health conditions.

## The Impending Storm: Addressing the Health Needs of Aging Populations

The perfect storm is brewing. The proportion of the world's population age 60 and older is projected to grow from 11% to 22% between the years 2000 and 2050, an absolute increase from 605 million to 2 billion people [1]. Health systems across the globe are ill prepared to meet the needs of aging populations. The challenges are many. Underinvestment in prevention contributes to the rising burden of chronic illness. Quality of care for chronic conditions is suboptimal; care for geriatric conditions such as falls or dementia is even worse [2]. Most older adults have multiple chronic conditions, but quality improvement efforts commonly focus on single diseases such as heart failure or diabetes. Older adults often see multiple providers in multiple settings of care (e.g., ambulatory primary and specialty care, hospital care, home care) and may have complex social needs. The health care they receive tends to be fragmented and poorly integrated with community services. The workforce lacks necessary geriatric competencies [3]. There is a pressing need to reengineer health systems to optimize health outcomes among older adults.

## Improving Quality and Outcomes of Care

Kenneth Coburn and colleagues report the impact on mortality at 5 years for a randomized controlled trial of a nurse case management intervention for US Medicare beneficiaries by Health Quality Partners (HQP), a non-profit quality improvement organization [4]. The study contributes to a complex and contradictory body of literature on improving efficiency, quality, and outcomes of care for elders with complex chronic illnesses. They found a 25% reduction in mortality in the intervention group compared to controls. HQP was one of only two out of 15 models of care coordination evaluated through the Medicare Coordinated Care Demonstration (MCCD) that had positive health outcomes [5]. Nevertheless, despite a meaningful reduction in mortality, the intervention was not an unqualified success. Findings were mixed for other HQP outcomes examined in the trial and reported elsewhere [5],[6]. At 2 years, there was no improvement in quality of life [5]. The overall intervention did not reduce hospitalization rates and was not cost saving. It was, however, cost saving for high risk participants, among whom there was a 39% reduction in hospitalizations and 37% reduction in emergency visits. Results for quality improvement measures were modest at best; there was improvement on four of 12 quality indicators assessing preventive services and only one of nine assessing preventable adverse outcomes. Patient and provider satisfaction was high [6].

The study had several limitations. Less than half of those eligible to participate did so. Study participants were younger, overwhelmingly white, and more educated and affluent than participants at other MCCD sites. Nonetheless, the study adds to a body of literature showing that multi-faceted interventions can improve health outcomes among chronically ill elders [7]. However, most successful interventions have occurred in the context of organized systems of care. There is little evidence on how to improve care among small independent primary care practices that lack the resources of larger organizations to implement intervention components. Kenneth Coburn and colleagues demonstrated that it is possible to provide support to small practices to improve health outcomes.

Why did HQP succeed when many have failed? Despite evidence for effective models of care, including interventions in socioeconomically disadvantaged communities [7],[8], well designed trials and large scale demonstration projects have often had disappointing results [9],[10]. The formal MCCD evaluation identified elements attributed to success: effective engagement of patients and providers; evidence-based patient education and self management support; management of care transitions; enhanced communication between providers; and effective medication management.

## Averting the Storm

Will we be able to avert the impending storm? There is an urgent need to do so. Without the concerted effort of policy makers and providers across multiple sectors, a disaster looms. While the challenges are many, the opportunities are enormous. Better quality of care for chronically ill older adults has been associated with improved functional status and reduced mortality [11],[12]. The paucity of evidence on how to reengineer health systems to reproducibly improve outcomes, or on how to adapt and scale successful models of care cannot be an excuse for inaction.

Several fundamental steps can foster progress. We can systematically learn from past successes and failures. Learning networks across communities and systems of care can support accelerated learning for the development, implementation, and adaptation of effective interventions [13]. Rigorous evaluations such as the one reported here should become routine. It is necessary to learn what works for which populations in which systems of care and which social and cultural contexts. New meaningful measures of quality for complex chronically ill adults are needed to better assess effectiveness.

For many reasons, traditional randomized controlled trials cannot provide all the needed evidence for interventions aimed at large scale health system transformation. The effectiveness of improvement interventions is dependent on contextual factors as well as the fidelity of implementation. Furthermore, interventions may evolve over time as learning occurs. Additional evidence can be derived from pragmatic trials designed to assess effectiveness in diverse practices in diverse communities; mixed method studies; and realist evaluations that assess the influence of contextual factors on outcomes and can provide insight into “what works, for whom, in what settings of care" [14]. To build the evidence base, an investment in research will need to accompany investments in health system redesign.

Caution is required. Because complex interventions are most successful in high risk populations, there is the possibility that resources will be targeted primarily to these highest cost users of health services, perpetuating underinvestment in chronic disease prevention and management across risk strata. We need to learn how to efficiently tailor services and interventions across the continuum of risk. Ultimately, the goal should be to reduce the population burden of chronic illness. This can only be accomplished by targeting the root causes of disease in the social determinants of health and an enhanced focus on prevention. Health system sustainability is dependent on improving the health of aging populations.

## Abbreviations

HQP: Health Quality Partners
MCCD: Medicare Coordinated Care Demonstration
